# Severe Vitamin B12 Deficiency Presenting as Pancytopenia, Hemolytic Anemia, and Paresthesia: Could Your B12 Be Any Lower?

**DOI:** 10.7759/cureus.29225

**Published:** 2022-09-16

**Authors:** Mary M Pelling, Stephen T Kimura, Erica J Han, Yoo Mee Shin

**Affiliations:** 1 Internal Medicine, Emory University, Atlanta, USA; 2 Internal Medicine, Emory University School of Medicine, Atlanta, USA; 3 Hospital Medicine, Emory University Hospital Midtown, Atlanta, USA

**Keywords:** megaloblastic anemia, pancytopenia, vegan, vitamin b12 deficiency, cobalamin, vitamin b12

## Abstract

Although severe vitamin B12 deficiency is rare in the United States, recent increases in the adoption of vegan lifestyles have led to a significant rise in the rates of B12 deficiency, along with its hematologic and neurologic sequelae, the latter of which is often irreversible.

We describe a case of a 39-year-old male who presented with a several-month history of progressively worsening word-finding difficulties, shortness of breath, and a four-day history of bilateral hand numbness and tingling. Laboratory data revealed pancytopenia with profound anemia. Markers of hemolysis were positive, including elevated indirect bilirubin, disproportionately elevated lactate dehydrogenase (LDH), low haptoglobin, negative direct anticoagulant test, and hypoproliferative reticulocyte index. Blood smear revealed hypersegmented neutrophils and macrocytosis. Vitamin B12 levels were undetectable, and anti-intrinsic factor and parietal cell antibodies were negative. A thorough history revealed a 20-year history of strict veganism without B12 supplementation. He was transfused with packed red blood cells and started on subcutaneous B12 injections with rapid improvement of his symptoms.

Early recognition of B12 deficiency causing the constellation of pancytopenia, hemolytic anemia, and neurologic symptoms is vital in preventing irreversible neurologic sequelae. This case also highlights the importance of accurate history taking to aid in early diagnosis of B12 deficiency, especially in the context of rising rates of veganism in the United States.

## Introduction

Vitamin B12, also known as cobalamin, is found in animal products, including fish, meat, poultry, eggs, and dairy. In the United States, vitamin B12 deficiency has been observed in 6% of adults under the age of 60, increasing to 20% among adults 60 and older [1]. Men and women are at equal risk for B12 deficiency, and white Americans are significantly more likely to be B12 deficient compared to African American and Hispanic/Latino individuals [2]. The most common causes of vitamin B12 deficiency include difficulty absorbing vitamin B12 from food, lack of intrinsic factor due to pernicious anemia, prior gastrointestinal surgery, prolonged use of certain medications such as metformin and proton pump inhibitors, and dietary deficiency [2]. B12 deficiency is more common among individuals over 60 years of age due to the increased prevalence of pernicious anemia, atrophic gastritis, and infection with *Helicobacter pylori *[1]. 

As of 2021, 3% of Americans adhere to a vegan diet [3], which entails eating foods that are only plant-based, while abstaining from all food produced from or by animals, including all meats, eggs, cheese, milk, and honey [4]. While there are many health benefits from adhering to a vegan diet, including a lower likelihood of developing elevated blood pressure, diabetes, high cholesterol, heart disease, and some types of cancer, there are also some important considerations: vegan diets can be associated with some key deficiencies including protein, calcium, omega-3 fatty acids, zinc, vitamin B12, and vitamin D [5]. Therefore, it is important to have proper supplementation when adhering to such a diet. One study looking at vitamin B12 levels in vegans residing in the United States revealed that after 4.2 years of veganism, 12% had low vitamin B12 serum levels, defined as <150 pmol/l [6]. A German study also looking at B12 levels in those following a strict vegan diet found that 58.3% had a B12 level <150 pmol/l after an average of 7.7 years [7].

Adverse outcomes of B12 deficiency can manifest as hematologic or neurologic consequences. A hallmark sign of B12 deficiency is megaloblastic anemia, classically found with hypersegmented neutrophils on peripheral blood smear. It is commonly seen with low counts of white blood cells, red blood cells, platelets, a combination of these, or (rarely) pancytopenia and hemolysis. Neurological changes associated with B12 deficiency include sensory deficits, paresthesia, weakness, ataxia, and gait disturbance, while severe cases can cause spasticity and paraplegia. Subacute combined degeneration is the most feared neurologic sequela and is characterized by demyelination of the dorsal, lateral, and spinocerebellar tracts of the spinal cord, which can lead to loss of fine touch, vibratory, and pressure sensation; weakness; visual impairment; changes in mental state; bilateral spastic paresis or paralysis; and progressive paresthesias. In pregnant and breastfeeding women, vitamin B12 deficiency can lead to neural tube defects, developmental delays, failure to thrive, and anemia in the offspring. Other systemic symptoms include glossitis, fatigue, palpitations, pale skin, weight loss, and infertility.

This article was previously presented as a meeting abstract at the Society of General Internal Medicine Annual Meeting in April 2022, as well as the Society of Hospital Medicine: Converge Conference in April 2022.

## Case presentation

A 39-year-old male with no significant past medical history presented with four days of a constant “pins and needles” sensation located from fingertips through elbows bilaterally as well as progressive shortness of breath and fatigue. He also reported feeling unbalanced and light-headed. Additionally, for the past seven months, he had been experiencing worsening confusion, including word-finding difficulties. A review of systems was positive for subjective and unintentional weight loss, night sweats, intermittent and blurry vision, and darkening of the patient’s urine. Further history revealed a strict vegan diet for 20 years with no vitamin supplementation. He denied any tobacco or drug use and drank alcohol only occasionally. 

Vital signs were notable for blood pressure 85/48 mmHg, heart rate 60 bpm, and normal oxygen saturation. Physical exam showed sublingual pallor and slow conversational speech but was otherwise normal. Laboratory results (Table 1) revealed pancytopenia with white blood cell count 1.7 k/mcL, hemoglobin 4.5 gm/dL, mean corpuscular volume (MCV) 103 fL, and platelets 111 k/mcL. Indirect bilirubin was elevated 1.74 mg/dL with a low reticulocyte index 0.1, elevated lactate dehydrogenase >3600 unit/L, and low haptoglobin < 3 mg/dL, but a negative direct anticoagulant test. Blood smear revealed hypersegmented neutrophils as well as marked macrocytosis. Vitamin B12 level was undetectable at <50 pg/mL but folic acid was normal at 16.2 ng/mL. Anti-intrinsic factor and parietal cell antibodies were negative. Laboratory values at presentation and two-month follow-up are presented in Table 1 and a case timeline in Table 2.

**Table 1:** Patient Laboratory Values Upon Admission and at Follow-up 2 Months Later Notes: (H) = high; (L) = low.

Hematology	Admission: July 2021	Follow-up: September 2021
White blood cells	1.7 k/mcL (L)	3.0 k/mcL (L)
Hemoglobin	4.5 gm/dL (L)	13.8 gm/dL
Mean corpuscular volume (MCV)	103 fL (H)	88.5 fL
Platelet count	111 k/mcL (L)	243 k/mcL
Hemolysis Studies		
Reticulocyte index	0.1 (L)	--
Bilirubin total	2.1 mg/dL (H)	0.7 mg/dL
Bilirubin indirect	1.74 mg/dL (H)	--
Haptoglobin level	<3 mg/dL (L)	--
Lactate dehydrogenase	>3600 unit/L (H)	--
Autoimmune Studies		
Direct antiglobulin test	Negative	--
Intrinsic factor antibody	Negative	--
Parietal cell antibody	Negative	--
Vitamin Levels		
Folate level	16.2 ng/mL	--
Vitamin B12 level	<50 pg/mL (L)	327 pg/mL

**Table 2:** Case Timeline

Hospital Day	Significant Events
-7 months	Onset of patient confusion including word finding difficulties, start of progressively worsening shortness of breath and fatigue
-4 days	Patient begins experiencing constant “pins and needles” sensation located from fingertips through elbows bilaterally
0	Patient presents in the emergency department and is admitted Vital signs notable for blood pressure 85/48 mmHg, heart rate 60 bpm, and normal oxygen saturation Physical exam showed sublingual pallor and slow conversational speech
+1 day	Demonstrated improvement in mental status, including word-finding difficulties
+2 day	Continued improvement including ataxia, fatigue, and shortness of breath. Discharged from hospital.
+ 1 month	Confusion completely dissipated. Some residual left-hand paresthesia.
+ 2 months	Hematologic labs showed improvement. B12 level normalized.

A comprehensive differential was formed including malignant (leukemia, lymphoma, myelodysplastic syndrome), infectious (HIV, hepatitis, viral etiologies, syphilis), autoimmune (systemic lupus erythematosus [SLE], pernicious anemia, autoimmune gastritis), hematologic (hemophagocytic lymphohistiocytosis), nutritional (B12 and folate deficiency), and endocrine (hypothyroidism) etiologies. A thorough workup included syphilis, HIV, viral hepatitis panel, Cytomegalovirus, Epstein-Barr virus, Parvovirus, and SLE, all of which were unremarkable. Intrinsic factor and parietal cell antibodies were negative. Thyroid stimulating hormone was slightly increased to 8.27 mcIU/mL with mildly low triiodothyronine 71 ng/dL and normal free thyroxine. Computerized tomography (CT) head scan without contrast was unrevealing with no acute abnormalities noted. Abdominal and pelvis CT with IV contrast was also unrevealing, with only a small hypodensity in the right hepatic lobe, making malignancy less likely. The patient was found to have a severe B12 deficiency, which offered a cohesive explanation for this patient’s symptoms. Therapeutic interventions for B12 deficiency led to rapid improvement, leading to the diagnosis.

During the patient’s hospital stay, he received three units of packed red blood cells and two subcutaneous doses of 1000 mcg B12 with rapid improvement in mental status, ataxia, and paresthesia. The patient was discharged with five more doses of Vitamin B12 1000 mcg/mL injectable solution to use daily for a total of seven consecutive daily doses. He was instructed to administer a dose once weekly for four weeks and then once monthly. He was discharged with hematologic and primary care follow-up appointments.

At the 1-month follow-up, the patient’s confusion had resolved and only mild residual left-hand paresthesia remained. After two months, all his hematologic and other relevant labs improved and his B12 level normalized. White blood cell count was 3.0 k/mcL, which was improved but still low; hemoglobin was 13.8 gm/dL with an MCV of 88.5 fL, and platelets were 243 k/mcL. Vitamin B12 level was 327 pg/mL.

## Discussion

Anemia related to vitamin B12 (cobalamin) deficiency is most frequently due to pernicious anemia or prior gastrointestinal surgery and much less commonly results from dietary deficiency. However, with increasing rates of veganism and vegetarian diets, B12 deficiency prevalence has been increasing. Vitamin B12, found primarily in animal products, is an important cofactor in DNA and RNA synthesis and tissues undergoing rapid cell turnover are most affected by this deficiency. A deficiency leading to clinical consequences typically takes one to four years to develop, with hematologic and neurologic symptoms being the most evident [8].

Hematologic consequences include macrocytosis, hypersegmented neutrophils, leukopenia, thrombocytopenia, and rarely, pancytopenia; in fact, pancytopenia, in which all blood cell lines are decreased, is seen in only 5% of patients with a known B12 deficiency [9]. Typically, with profound anemia as exhibited in this patient, tachycardia would be an expected associated vital sign, but in this case was normal, likely due to the chronicity of his disease state. Furthermore, evidence of hemolysis in these patients is seen in 1.5% of cases, making this presentation a rare manifestation of severe vitamin B12 deficiency [9]. Hemolysis in B12 deficiency most often occurs within the bone marrow, often referred to as intramedullary hemolysis or ineffective erythropoiesis. This occurs when poorly formed erythropoietic cells are phagocytosed within the bone marrow. Few blood cells are then able to exit the bone marrow to enter systemic circulation, which manifests as a low reticulocyte count or index. It is consistent with a hypoproliferative state and poor bone marrow response, as demonstrated in this patient. Extramedullary hemolysis has also been rarely reported secondary to a variety of factors, most often via antibody-mediated destruction, which was negative in this patient. Other reported causes of hemolysis include high levels of homocysteine leading to endothelial dysfunction and lysis, or due to mechanical destruction of poorly-formed red blood cells as they traverse small capillaries beds [10].

B12 myeloneuropathy commonly affects white matter in the dorsal and lateral columns of the spinal cord, which are responsible for the conduction of vibratory and positional sense. Subacute combined degeneration is the most feared neurologic sequelae and is characterized by demyelination of these dorsal and lateral columns. Common effects include sensory deficits, paresthesia, weakness, ataxia, mental status changes, and gait disturbance; severe cases of B12 deficiency can lead to irreversible damage, spasticity and paraplegia [11].

After treatment with supplemental B12, anemia usually completely resolves in four to six weeks. Recovery from neurologic symptoms can take several months after supplementation and in some cases symptoms are permanent. Therefore, it is imperative to start treatment to replenish B12 when clinical suspicion is high. 

While our clinical suspicion was high for a vitamin B12 deficiency, given the patient's dietary history and clinical symptoms, it was important to consider a broad differential of alternative etiologies such as malignancy, rheumatologic, and infectious in the preliminary workup.

## Conclusions

This paper describes a severe case of vitamin B12 deficiency presenting with the unique constellation of cognitive decline, paresthesias, pancytopenia, and hemolytic anemia in a member of a demographic group in which this condition is rarely observed. Ultimately, the key history finding of a strict vegan diet and unrevealing secondary workup allowed for the diagnosis and treatment of vitamin B12 deficiency. Fortunately, our patient’s symptoms of mental fog, ataxia, and paresthesia improved rapidly with supplementation and laboratory abnormalities were all improved and normalized by two months after discharge. This case highlights the importance of taking a thorough history, including dietary habits, when hematologic and neurologic symptoms may indicate a vitamin deficiency. It also highlights the importance of vitamin B12 supplementation for patients following a strict vegan or vegetarian diet. 
